# Pan-Angiosperm Analysis of the CLE Signaling Peptide Gene Family Unveils Paths, Patterns, and Predictions of Paralog Diversification

**DOI:** 10.1093/molbev/msaf294

**Published:** 2025-11-13

**Authors:** Iacopo Gentile, Miguel Santo Domingo, Sophia G Zebell, Blaine Fitzgerald, Zachary B Lippman

**Affiliations:** Cold Spring Harbor Laboratory, Cold Spring Harbor, NY, USA; Howard Hughes Medical Institute, Cold Spring Harbor Laboratory, Cold Spring Harbor, NY, USA; Cold Spring Harbor Laboratory, Cold Spring Harbor, NY, USA; Howard Hughes Medical Institute, Cold Spring Harbor Laboratory, Cold Spring Harbor, NY, USA; Cold Spring Harbor Laboratory, Cold Spring Harbor, NY, USA; Howard Hughes Medical Institute, Cold Spring Harbor Laboratory, Cold Spring Harbor, NY, USA; Cold Spring Harbor Laboratory, Cold Spring Harbor, NY, USA; Howard Hughes Medical Institute, Cold Spring Harbor Laboratory, Cold Spring Harbor, NY, USA; Cold Spring Harbor Laboratory, Cold Spring Harbor, NY, USA; Howard Hughes Medical Institute, Cold Spring Harbor Laboratory, Cold Spring Harbor, NY, USA

**Keywords:** small signaling peptides, CLE genes, gene annotation, graph embedding, redundancy, paralog evolution, protein evolution

## Abstract

The compositions of conserved gene families often vary widely between species, complicating predictions and experimental tests of shared versus distinct functions, especially in families shaped by extensive duplication, redundancy, and paralog diversification. The plant *CLV3/EMBRYO-SURROUNDING REGION* (*CLE*) small signaling peptide family exemplifies these challenges. Although genetic studies in model systems have identified shared roles for a few *CLE* genes and species-specific redundancies, an evolutionary analysis of the entire family over deep time could empower predictive and experimental dissections of functions obscured by redundancy. We developed a scanning pipeline that de novo annotated *CLE* genes from 2,000 genomes representing 1,000 species, uncovering thousands of previously undetected family members and producing a comprehensive view of the family's evolution and sequence diversification over 140 million years. Computational modeling of coding and cis-regulatory regions predicted lineage-specific asymmetries in paralog redundancy, stemming from ancestral amino acids in the functional core of the dodecapeptide and partial conservation of promoter elements. We tested these predictions using two genome-editing strategies in Solanaceae. Base-editing of deeply conserved residues in the CLV3 dodecapeptide and its paralogs across three species confirmed their critical roles in repressing stem cell proliferation, and multiplex CRISPR knockouts of the 52 tomato *CLE* genes resolved simple and complex redundancies, revealing previously uncharacterized regulators of shoot architecture and plant size. These findings show how both peptide and cis-regulatory erosion shape *CLE* redundancy and provide a framework for detecting and translating deep evolutionary signals into testable genetic hypotheses across compositionally complex gene families.

## Introduction

High-throughput genome sequencing and computational genomics have transformed our understanding of gene family evolution across evolutionary timescales. Comparative analysis of genome composition has revealed dynamic and complex patterns of gene birth, death, and functional divergence. Gene families, formed and expanded through duplication events, exhibit remarkable variation in sequence, expression, and function across both distantly and closely related species (Nei 2013; Murat et al. 2012). The mechanisms driving this diversity operate through distinct evolutionary trajectories: Initial redundancy following a duplication event typically degrades through mutational drift, often resulting in gene loss (pseudogenization). However, through mutational serendipity and under certain selective pressures, a duplicated gene (hereafter, paralog) may partition its functions with its ancestor (subfunctionalization) or acquire new roles (neofunctionalization) (Wagner 2008; Dittmar and Liberles 2011). Although these classical long-term endpoints of paralog functional evolution have been well documented across many lineages, the evolutionary trajectories and dynamics of paralogous gene diversification over shorter timescales are less understood (Lynch et al. 2001; Lynch and Conery 2003; Birchler and Yang 2022). Recent pan-genomic studies, spanning both single species and multiple species in lineages spanning genera and families, offer opportunities to capture a range of evolutionary timescales that can reveal how lineage-specific duplications diversify gene families in sequence and function (Shang et al. 2022; Lian et al. 2024; Khan et al. 2024; Liu et al. 2024; Benoit et al. 2025; He et al. 2025; Wang et al. 2025; Cheng et al. 2025). In particular, deep evolutionary sampling through pan-genomics can reveal how lineage- and species-specific paralog redundancies arise and shift from the combined effects of coding and noncoding sequence variation (Kwon et al. 2022; Light and Kraulis 2004; Tvrdik and Capecchi 2006).

Following whole-genome or local gene duplication events, redundancy among paralogs allows mutations to accumulate in coding and regulatory sequences, leading to unpredictable changes in initial redundancy relationships that can affect genotype–phenotype relationships. An integrated approach that combines comprehensive phylogenetic sampling using expansive pan-genomic data with predictive computational modeling via machine learning approaches has the potential to reveal the dynamics of how redundancy relationships evolve to shape gene families and their functional compositions. A remaining barrier, however, is incomplete and inconsistent gene annotations between reference genomes, which continues to obscure the full extent of genetic and allelic diversity, particularly in gene families that have undergone, and continue to undergo, frequent duplication and sequence evolution.

A striking example of these challenges is found in small signaling peptide gene families (Furumizu and Sawa 2021; Furumizu and Aalen 2023; Furumizu and Shinohara 2024; Chang and Xiao 2025). Among the many examples documented, the plant *CLAVATA3/EMBRYO SURROUNDING REGION-RELATED* (*CLE*) family represents one of the most extreme examples. *CLE* genes encode approximately 100 amino acid peptide sequences that are proteolytically processed into 12-amino acid small signaling peptides (dodecapeptides). The dodecapeptides are glycosylated and then secreted to bind leucine-rich repeat receptor-like kinases (LRR-RLKs) on the surface of neighboring cells. These interactions mediate downstream signaling events that are critical for diverse developmental and physiological programs (Whitewoods 2021). However, high sequence divergence surrounding the functional dodecapeptides coupled with extensive copy number variation and challenges in detecting tissue- and cell-specific expression have confounded CLE family annotation and thus predictions that could permit systematic and comprehensive comparative functional analysis within and across species, especially paralog redundancy (Carbonnel et al. 2022, 2023). Indeed, a comprehensive mutational analysis of all *CLE* genes in the model *Arabidopsis thaliana* (hereafter *Arabidopsis*) revealed that most single-gene knockouts show no obvious phenotypes, suggesting widespread redundancy and compensatory relationships across family members that can only be revealed through high-order genetics (Yamaguchi et al. 2017), as demonstrated in the meristem interactive signaling between tomato *SlCLV3* and *SlCLE9* (Rodriguez-Leal et al. 2019) and *Arabidopsis AtCLV3*, *AtCLE16*, and *AtCLE17* (Dao et al. 2022).

Here, we aggregated plant pan-genomic resources and developed a computational pipeline to rapidly identify and annotate *CLE* genes across 2,000 genomes representing 1,000 species and spanning 140 million years of evolution (De Bodt et al. 2005). By integrating comparative phylogenetic analysis spanning ancient and recent evolutionary timescales, predictive computational modeling of the mutational landscape, and functional characterization through CRISPR genome editing, we uncovered mechanisms underlying the maintenance and diversification of *CLE* paralog redundancy. Our findings demonstrate that resolving the long-term dynamics of coding and regulatory sequence evolution among gene family members can predict the architectures of complex paralog interactions, exposing redundancy relationships and enhancing the predictability of genome-editing outcomes.

## Results and Discussion

### Pan-Angiosperm Discovery and Analysis of the CLE Peptide

**Fig. 1.**
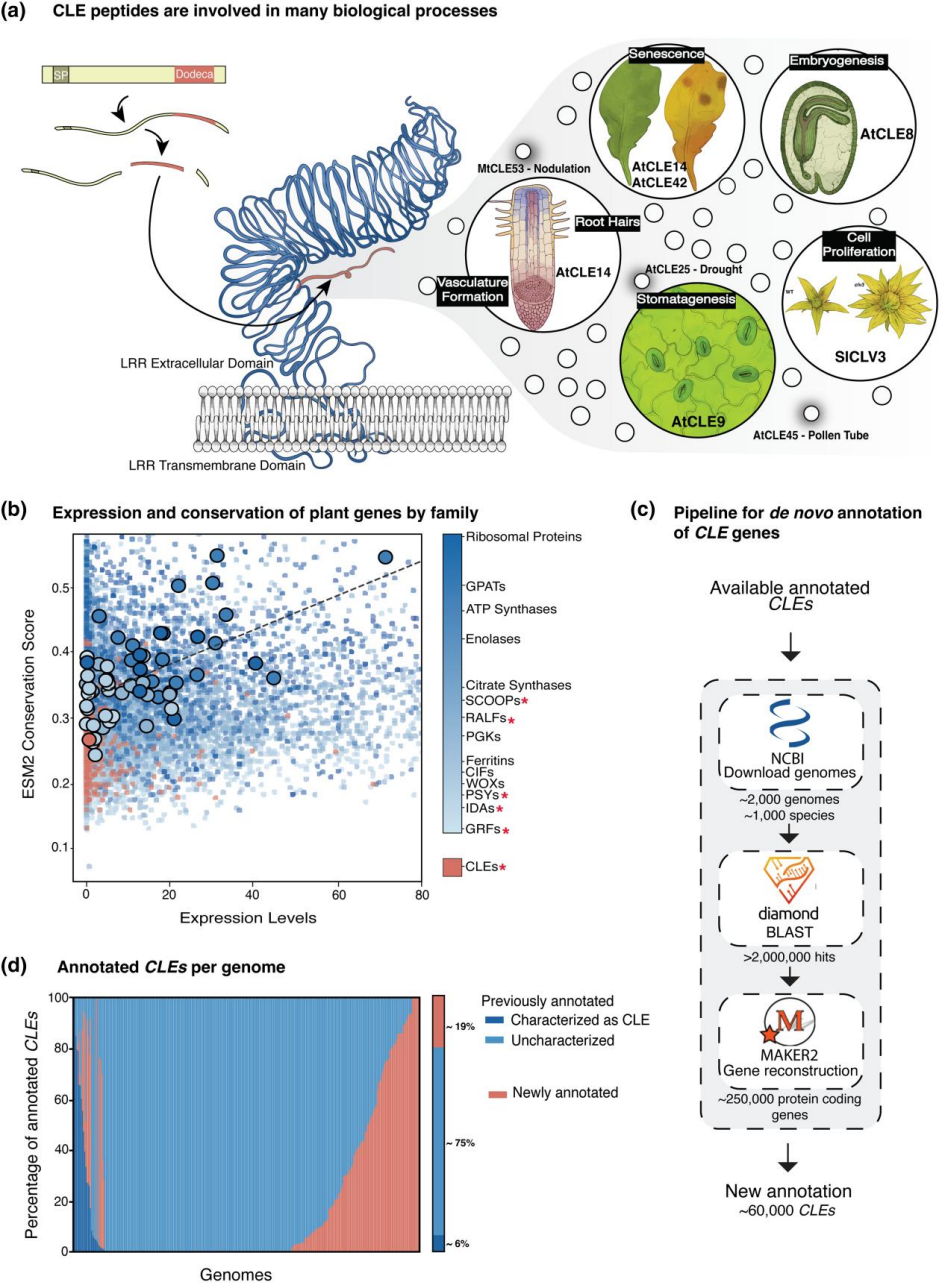
De novo annotation of CLE genes finds 60,000newly annotated CLE genes in 2,000 analyzed plant genomes. (a) CLE genes are post-translationally modified into active dodecapeptides, which are bound by LRR-RLKs as a part of signaling in diverse plant developmental processes (some examples are represented in the different bubbles). (b) Low conservation score derived from the EMS2 language model and low expression of CLE genes (red) compared to other gene families. * indicates small signalling peptide families. (c) This study's pipeline to annotate CLE genes, consisting of a first step with Diamond, followed by gene reconstruction by MAKER2 and filtering. (d) Percentage of newly annotated (red) or reannotated *CLE* genes in a subset of species with annotated genomes. Reannotated *CLE* are further stratified in previously known (dark blue) and previously uncharacterized (light blue). Aggregated data across genomes are also summarized and percentage values are shown.

*CLE* genes encode precursor proteins typically less than 100 amino acids in length that consist of an N-terminal Golgi signal peptide, a variable domain with significant sequence divergence, and a C-terminal 12-amino acid CLE motif called a dodecapeptide (Fig. 1a; Fletcher 2020). After proteolytic cleavage and release from the precursor, some CLE dodecapeptides undergo post-translational modifications for their activity (Whitewoods 2021; Jeong et al. 2025; Ohyama et al. 2009). By binding to and signaling through leucine-rich repeat (LRR) receptors, these small signaling peptides regulate numerous critical developmental and physiological programs, both conserved and species specific (Fletcher 2020; Araya, Von Wirén, and Takahashi 2014). For instance, *CLV3* is a deeply conserved *CLE* family member that represses stem cell proliferation in the shoot apical meristem (Fletcher et al. 1999; Benoit et al. 2025; Rodriguez-Leal et al. 2019; Kwon et al. 2022), whereas the *Arabidopsis AtCLE14* and *AtCLE42* paralogous genes regulate leaf senescence (Zhang et al. 2022a,b), and *MtCLE53* in the leguminous species *Medicago truncatula* controls root nodulation (Karlo et al. 2020) (Fig. 1a).

Beyond many past and present efforts to elucidate the functional roles of individual CLE peptides, a long-standing, yet intractable, endeavor has been to study the complex evolutionary histories that have shaped *CLE* family compositions and functions, as this understanding may provide insights into the dynamics and mechanisms driving functional diversification. Highly variable *CLE* family compositions, rapid coding sequence divergence, and generally low expression levels all complicate annotation efforts (Carbonnel et al. 2022). In particular, resolving *CLE* family sequence evolution has been hampered by the difficulties associated with aligning short, rapidly evolving proteins, often yielding low-quality alignments that compromise downstream analyses (Goad et al. 2017).

The exponential increase in reference genomes along with alternatives to conventional sequence alignment approaches has opened opportunities to investigate *CLE* family diversification. As a first step to improve *CLE* annotation, we adopted a method to assess and compare sequence conservation among members and between gene families based on the protein language model EVOLUTIONARY SCALE MODELING 2 (ESM2) (Yeung et al. 2023), bypassing the need for multiple sequence alignment. We first validated that ESM2 was appropriately trained for this task by confirming its ability to detect fundamental elements—such as the dodecapeptide (Figure S1a). We then applied it to the current repertoire of annotated gene families across plant genomes, which revealed that *CLE* genes and other small signaling peptides exhibit much lower conservation and expression compared to other gene families. For example, ribosomal proteins and core metabolism genes display the highest conservation, whereas *CLEs* are more closely associated with other small signaling peptides and specific transcription factor families (Fig. 1b). These observations point out the fast-evolving nature of signaling peptides, such as GROWTH-REGULATING FACTORS (GRFs), PLANT PEPTIDE CONTAINING SULFATED TYROSINE (PSY), and CASPARIAN STRIP INTEGRITY FACTORS (CIFs), with one exception being SERINE-RICH ENDOGENOUS PEPTIDE INDUCERS (SCOOPs), perhaps owing to them being Brassicaceae specific and having a narrower evolutionary scale (Snoeck et al. 2024).

This ESM2 analysis validates the extreme diversification of the *CLE* gene family, underscoring the annotation challenges inherent to these genes and likely other small signaling peptide families. The more rapid evolutionary dynamics compared to other families and restricted expression profiles of *CLE* genes suggest that the annotation challenges previously observed in models such as *Arabidopsis* and tomato (*Solanum lycopersicum*) (Carbonnel et al. 2022, 2023) are widespread among other plant genomes. In addition, general annotation tools are more likely to miss *CLE* genes due to their short sequences, limited transcriptional support, and low homology scores. To overcome these challenges, we developed a pipeline to comprehensively re-annotate existing and discover undocumented *CLE* family members via a pan-angiosperm scan of over 2,000 genomes spanning over 1,000 species, with melon, tomato, soy, maize, and *Arabidopsis* being among species with high representation with multiple accessions being represented (Figure 1c; Table S1; see Materials and Methods). We first compiled peptide data from 400 well-annotated species’ genomes (Table S1), originally collated from our Conservatory project that defines conserved noncoding sequences (CNSs) (Hendelman et al. 2021; Amundson et al. 2025), to create a dense homology search dataset. Using Diamond, a tBLASTn-like tool (Buchfink et al. 2015), we scanned genomic regions for *CLE*-like sequences. We then directed the annotation algorithm MAKER2 (Holt and Yandell 2011) to those regions, increasing annotation sensitivity by reducing the search space. Finally, we used a hidden Markov model to enrich true *CLE* genes by confirming the presence of a signal peptide. This approach identified over 2 million BLAST hits, of which 250,000 were annotated as protein-coding genes by MAKER2, ultimately yielding 60,000 genes classified as *CLEs* (Fig. 1c).

To evaluate the impact and accuracy of our pipeline, we first applied it to a subset of well-annotated genomes (Hendelman et al. 2021; Amundson et al. 2025; see https://conservatorycns.com for details). We found that over 40% of these species harbored previously unannotated *CLE* genes, and nearly all species contained mis-annotated *CLE* genes (ie genes annotated as protein-coding but not recognized as *CLE* family members) (Fig. 1d; Table S2). In well-studied genomes such as *Arabidopsis*, all *CLE* genes were correctly annotated, whereas the close relative *Arabis alpina* had 14 family members that were not annotated, increasing the total number of *CLE* genes in this species to 34.

To further evaluate the scalability of our approach to other small peptides, we applied it to the *SCOOP* gene family that was also the subject of annotation-mining investigations (Yang et al. 2023). We recovered all predicted *SCOOPs* despite the original search dataset having been ablated so not all *SCOOPs* were present. By applying this pipeline to the genome of *Cardamine hirsuta* in the Brassicaceae family, which was not considered in the original study, we found eight unannotated *SCOOPs*. This highlights the potential of this pipeline to go beyond *CLE* genes and applied to other small peptide families (Table S3).

### *CLE* Gene Family Sequence Relationship and Modeling Coevolution With LRR Receptors

In addition to annotation challenges, the short sequences and extreme sequence diversification of *CLE* genes impede the construction of reliable multiple sequence alignments to build robust phylogenetic trees within and across species. Although current methods each have their own limitations in accurately decomposing the evolutionary history of this family, graphical representations of gene clusters based on reciprocal BLASTp networks have been shown to infer putative orthogroups (Goad et al. 2017). We attempted to use this approach (Goad et al. 2017); however, standard reciprocal BLASTp algorithms, such as CLANS (Frickey and Lupas 2004), are sensitive to sample size, even under approaches such as MMseqs2, which are less affected by this aspect (Steinegger and Söding 2017), typically capture only closely related relationships, making it challenging to decompose proximal similarity among clusters. We therefore employed *Node2Vec*, a graph-representation embedding method that enables efficient analysis while minimizing redundancy and noise by taking a random-walk approach (Grover and Leskovec 2016). The resulting latent space shows a less distorted organization with a more uniform separation among CLE peptides compared to CLANS-like methods characterized by highly localized clusters that are increasingly distant from each other (Figure S1b). The resulting high-dimensional *Node2Vec* embedding was then projected onto a two-dimensional map using PHATE (see Materials and Methods), thereby preserving both global and local data structures and overcoming the limitations of methods that capture only local relationships (Moon et al. 2019).

**Fig. 2.**
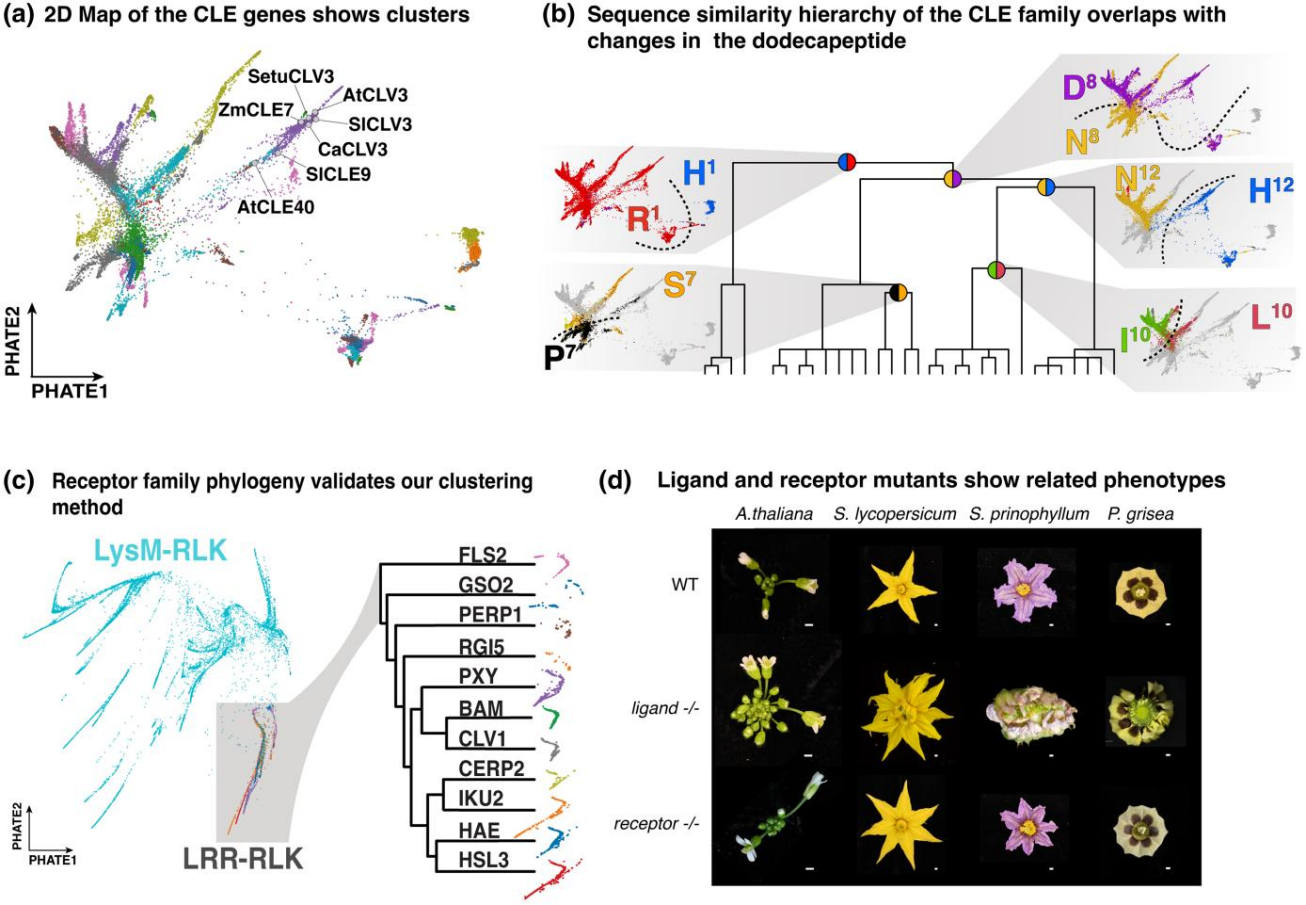
Our pipeline enables new analysis of the evolutionary dynamics of CLE genes. (a) PHATE map of the BLASTp pairwise comparison network of *CLE* genes showing different clusters, highlighting known *CLV3* orthologous and paralogous genes that are proximal to each other. (b) Dendrogram of hierarchical clustering of *CLE* genes showing evolutionary splits and nucleotide substitutions in the functional dodecapeptide driving them. For each major split, the PHATE map projections of specific amino acid changes at specific positions follow the branching pattern of the dendrogram (as shown based on the orientation of the colors in the circles located at each split point). (c) PHATE plot of BLASTp pairwise comparison network of LysM-RLK family genes, highlighting LRR-RLK clade and the correspondence of our clustering with previously published phylogenetic relationships. (d) Representative apical-meristem-derived floral development phenotypes of CLE ligand and receptor mutants in several species, together with WT. Ligand mutant corresponding to *Atclv3* in *A. thaliana*, *Slclv3 Slcle9* in *S. lycopersicum*, *Spriclv3a Spriclv3b* in *S. prinophyllum*, and *Pgclv3 Pgcle9* in *P. grisea*. The receptor mutant corresponds to *clv1* mutant in all species. Scale bar is 1 mm.

The resulting projection revealed distinct clustering patterns that reflect known relationships and give important context to newly annotated genes. For example, CLV3 and close paralogs such as SlCLE9 in tomato and AtCLE40 in *Arabidopsis* cluster together, demonstrating their sequence similarity (Fig. 2a;Table S4). Moreover, hierarchical clustering (see Materials and Methods) of the complete set of unprocessed CLE protein sequences produced a dendrogram that exhibits major splits associated with amino acid changes in the dodecapeptide (Fig. 2b). These splits reveal a parsimonious structure behind the clustering hierarchical structure that confirms the central contribution of dodecapeptide sequences on the overall patterns of full protein sequence divergence. In contrast, other sequence-specific features, such as the composition of the Golgi N-terminal signal sequence (Figure S2), showed a weaker association, reinforcing that the dodecapeptide is the dominant element shaping the observed patterns, owing to its deeply conserved role in signaling through cell-surface LRR receptors. We further evaluated the projected relationships derived from MMseq2 rather than BLASTp and resulted in similar comparable patterns (see Materials and Method; Figure S3).

Given the outcome from applying graph-representation embeddings, we next generated a vector representation of the *LEUCINE-RICH REPEAT RECEPTOR-LIKE* (*LRR-RLK*) gene family, which includes canonical *CLE* peptide receptors such as *CLAVATA1* (*CLV1*) and *BARELY ANY MERISTEM* (*BAM*) (Ogawa et al. 2008; Rodriguez-Leal et al. 2019; Seo et al. 2024). Leveraging the more reliable annotation status of this gene family, we searched for *LRR-RLK* genes in the same subset of 400 Conservatory genomes (see Materials and Methods). Cluster analysis revealed clear groupings consistent with previous studies (Man et al. 2023), with the embedding precisely distinguishing known clades such as *CLV1*, *BAMs*, and *PHLOEM INTERCALATED WITH XYLEM* (*PXY*) (Fig. 2c).

The functional relationship and molecular modes of action between CLE peptides and LRR-RLKs are well established, including detailed structural and biochemical analyses (Zhang et al 2016). Furthermore, comparative genetic studies across species have repeatedly demonstrated that interactions between members of these families are coevolutionarily stable (Je et al. 2018; Kwon et al. 2022; Rodriguez-Leal et al. 2019; Seo et al. 2024; Ogawa et al. 2008). For instance, the functions of orthologs of *CLV3* and its primary receptor *CLV1*—whose mutations cause stem cell overproliferation, increased meristem size, and floral organ overproliferation (fasciation)—are deeply conserved, spanning maize, *Arabidopsis*, tomato, and the Solanaceae species *Physalis grisea* (ground-cherry) (Ogawa et al. 2008; Rodriguez-Leal et al. 2019; Seo et al. 2024). We also leveraged genome editing in our recently established Solanaceae genetic system, forest nightshade (*Solanum prinophyllum*) (Benoit et al. 2025) to mutate its *CLV1* ortholog, which caused moderate fasciation like in tomato (Fig. 2d;Table S5).

Given this deeply conserved functional relationship, we sought to test whether the graph-based approach can be applied to examine coevolution between receptors and their peptide ligands. A recent study on the interaction between the signaling peptides SCOOPs and their receptor MIK2 showed the power of generative modeling in dissecting interaction mechanisms (Snoeck et al. 2024). Following this methodology, we applied modeling based on AlphaFold-Multimer to delineate the interaction landscape within the LRR binding region (Figure S4a). Among putative interacting positions, we observed that certain residues in the receptor domain showed a distribution of amino acid identity per position in the embedding space with clear splits, as observed for the dodecapeptide (see Materials and Methods). These graph-embedding-based observations suggest that these CLE peptide and LRR binding domain residues may have been involved in evolutionarily important ligand interactions, which cannot be defined by traditional methods that rely on paired multiple sequence alignments between known ligands and receptors (Bitbol 2018). A striking example of this relationship is positions 152 and 177, both having asparagine (N) in the CLV1 and BAM clades, but serine (S) in PXYs (Figure S4b). In a crystal structure, these residues are predicted to be important for interaction with position 1 of the CLE dodecapeptide, representing a restricted amino acid whose shift in residues mirrors the R–H dichotomy observed at position 1 in our dodecapeptide clustering (Zhang et al. 2016). In addition to validating the utility of a graph-representation embedding approach in comparative phylogenetics, this analysis provided insights into possible coevolution of CLE peptides and their receptors.

### Mutational Effect Analysis of CLE Dodecapeptides

Beyond assembling a comprehensive pan-angiosperm collection of *CLE* genes for cluster association, we asked whether uncovering the full breadth of the family's sequence diversity could yield additional insights by applying our dataset to an emerging area of computational genetics focused on identifying mutational effect (ME) signals embedded within natural variation. As sequences diverge, mutations appear and get fixed based on their individual codon distance and biochemical consequences of that mutation at the amino acid level, but also on their sequence context. To capture these effects, we applied a Potts model to learn the coevolutionary patterns among residues within peptides and predict the effects of mutations from deep sampling of sequence data (Riesselman, Ingraham, and Marks 2018; Hopf et al. 2017). Indeed, contact maps between amino acids in peptides derived from our model show a local pattern of coevolutionary behaviors between close amino acid neighbors, suggesting strong context position effects (Figure S5a). Importantly, these patterns strongly correlate [Spearman ρ = 0.465 {*P* = 2.02e-08}] with physical interaction of emulated physical interactions within the dodecapeptide (Lewis et al. 2025) (Figure S5b and c; see Materials and Methods).

**Fig. 3.**
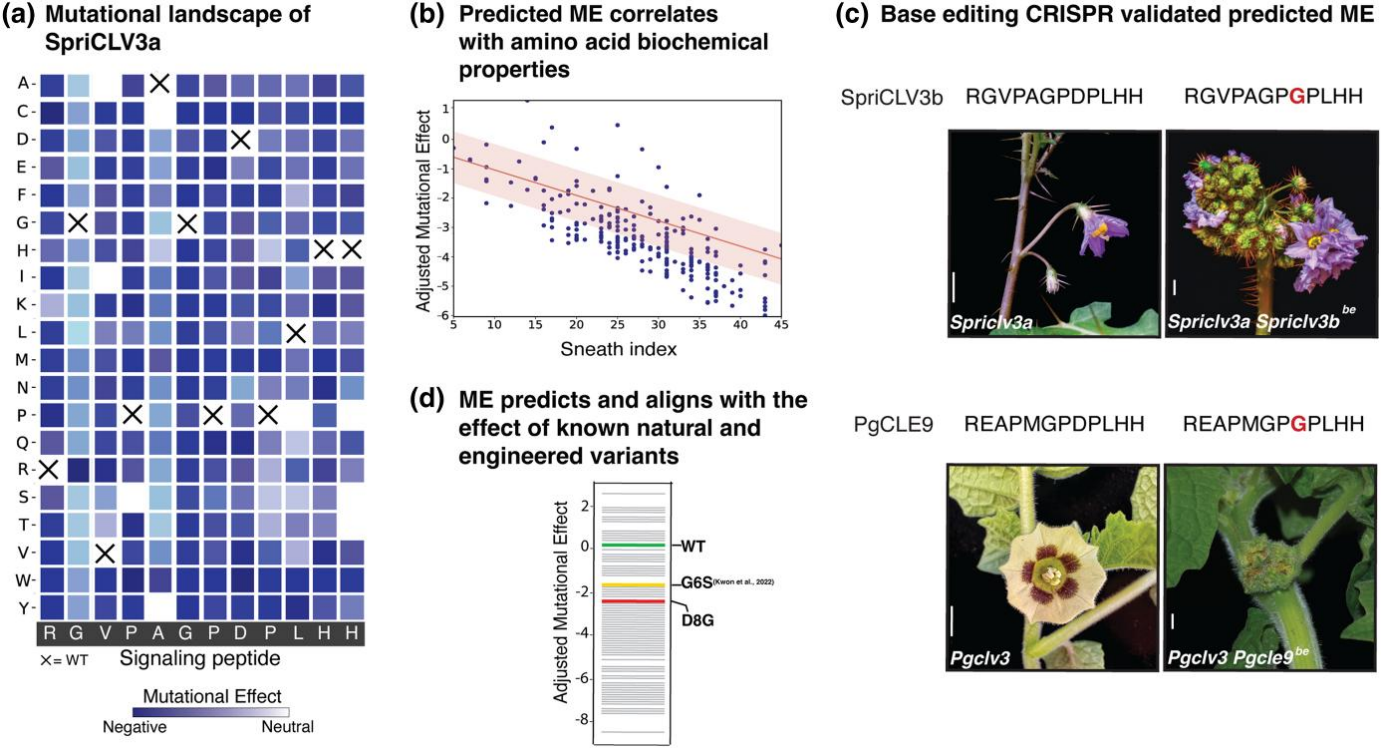
ME analysis of the newly expanded *CLE* family allows phenotype prediction. (a) Mutational landscape of *SpriCLV3* derived from the Potts model. Crosses show WT amino acid position. (b) Predicted ME correlates with the Sneath index, representing the dissimilarity of the biochemical properties of the amino acids. R^2^ = 0.54; MSE = 0.75; ρ = −0.086. (c) Dodecapeptide sequence and phenotypes of *CLV3*-clade double mutant base-edited (D8G) plants showing fasciation in two species (*S. prinophyllum* and *P. grisea*) compared to single *clv3* mutants. Base edited residue highlighted in red. (d) Perceived phenotype strength (WT, green; moderate, yellow; severe, red) of naturally occurring (G6S) and designed substitution (D8G) correlates with predicted ME.

Using forest nightshade *CLV3* (*SpriCLV3*) as a case study, we observed that its mutational landscape exhibits a nonuniform distribution of effects along the dodecapeptide (Fig. 3a). Notably, positions 2 and 5 (corresponding to glycine and alanine, respectively) demonstrate less deleterious MEs, suggesting a lower sensitivity to substitutions, consistent with findings in *Arabidopsis* (Kondo et al. 2006, 2008; Ogawa et al. 2008). To further validate this observation, we benchmarked our tomato SlCLV3–SlCLV1 ligand–receptor model against SSIPe, a hybrid model that combines sequence and structure profiling with force fields to calculate binding free-energy changes of protein–protein interactions (Huang et al. 2020), as well as against docking estimates generated by AlphaFold2, which have proven effective in predicting the impact of amino acid substitutions on docking (Yang, Milas, and White 2022). Initially, our analysis showed that the predicted MEs correlated with evolutionary signals derived from BLOSUM matrices (Figure S6a; R^2^ = 0.026; MSE = 5.43; ρ = 0.4). To test if biochemical signals are embedded in the Potts model, we regressed out the BLOSUM signal and observed strong correlations with both the biochemical properties of the substitutions (Sneath index—a measure of the average biochemical differences between amino acids) (Fig. 3b; R^2^ = 0.54; MSE = 0.75; ρ = −0.086) and with alterations in binding energy (Figure S6b; R^2^ = 0.88; MSE = 0.08; ρ = 0.90). Additionally, docking between SpriCLV3 and SpriCLV1 exhibited a moderate correlation with our ME estimates (R^2^ = 0.57; MSE = 0.82; ρ = 0.48) (Figure S6c) We further validated AlphaFold:Multimer with Alphafold3, showing a strong correlation between the two models (Figure S6d).

Following exhaustive in silico validation, we assessed functional predictions of our approach in vivo. Our previous work demonstrated that a naturally occurring amino acid substitution in SlCLE9 (the partially redundant paralog of SlCLV3) from glycine to serine at position 6 weakens peptide function, leading to only partial compensation for loss-of-function mutations in *SlCLV3* (Kwon et al. 2022; Aguirre et al. 2023). In contrast, in other Solanaceae, such as ground-cherry and petunia (*Petunia hybrida*), this residue is maintained as the ancestral glycine, and *CLE9* orthologs are more potent compensators when *CLV3* orthologs are mutated (Kwon et al. 2022). Notably, our modeling similarly predicted a deleterious effect associated with the serine substitution, corroborating our previous genetic findings (Fig. 3c).

To further test functional predictions from our model, we examined species with differing *CLV3* paralog diversifications. The ground-cherry *SlCLE9* ortholog (*PgCLE9*) retains near-complete redundancy with *PgCLV3*, whereas in forest nightshade, *SpriCLE9* was lost, but redundancy was restored via a local duplication of *SpriCLV3*. We performed CRISPR base-editing of *PgCLE9* in ground-cherry and of the derived *SpriCLV3b* paralog in forest nightshade within their respective *clv3* mutant backgrounds (Fig. 3c) (Kwon et al. 2022; Benoit et al. 2025). Substitution of glycine with serine at position 8 in both species' *CLV3* paralogs resulted in a severe fasciation phenotype, supporting another model prediction (Fig. 3d) and indicating that the amino acid change in this engineered allele exerts a stronger mutant phenotypic effect than the previously characterized hypomorphic G6S change in tomato (Fig. 3d). These analyses demonstrate the predictive power of functional variants through molecular evolution modeling, aligning both computational and empirical evidence.

### Assessing Mutational Burden in Predicted *CLE* Paralog Redundancy Detects Asymmetric Divergence

A current challenge in interrogating the functions of complex gene families via genome editing is the poor predictability of genotype–phenotype relationships among paralogs (Benoit et al. 2025; Iohannes and Jackson 2023). Building on the computational and experimental validation of our ME predictive model, we repurposed it to assess mutational burdens among *CLE* paralogs (see Materials and Methods; Figure S7a). Paralog evolution typically starts from complete redundancy, which relaxes selective pressures and permits the accumulation of mutations, eventually leading to divergence in sequence, expression behavior, and biochemical properties, thus potentially impacting multiple layers of biological function, including organismal phenotype. Mechanistically, this divergence can occur at both the coding and regulatory levels (Wagner 2008; Dittmar and Liberles 2011). Our model's ability to quantify MEs can offer new insights into the impact of protein-level variation. Given the difficulties in defining the products of gene duplication that retain functional relationships, we classified genes as functional paralogs based on both coding and promoter sequence conservation, where most cis-regulatory function is often found (see Materials and Methods) Using the paralog groups thus identified, we applied our Potts model to quantify specific paralogs that accumulated more deleterious mutations.

In a broader analysis, we compared the mutational burden of paralogous dodecapeptides versus their putative homologs while also accounting for duplication age based on synonymous substitutions. Our results indicate that closely related, derived paralogs of ancestral family members tend to accumulate more deleterious substitutions than nonparalogous genes (Figure S7b; see Materials and Methods). These findings are consistent with the theoretical expectation that gene duplication and redundancy allow greater mutation accumulation via relaxed selection (Wagner 2008).

**Fig. 4.**
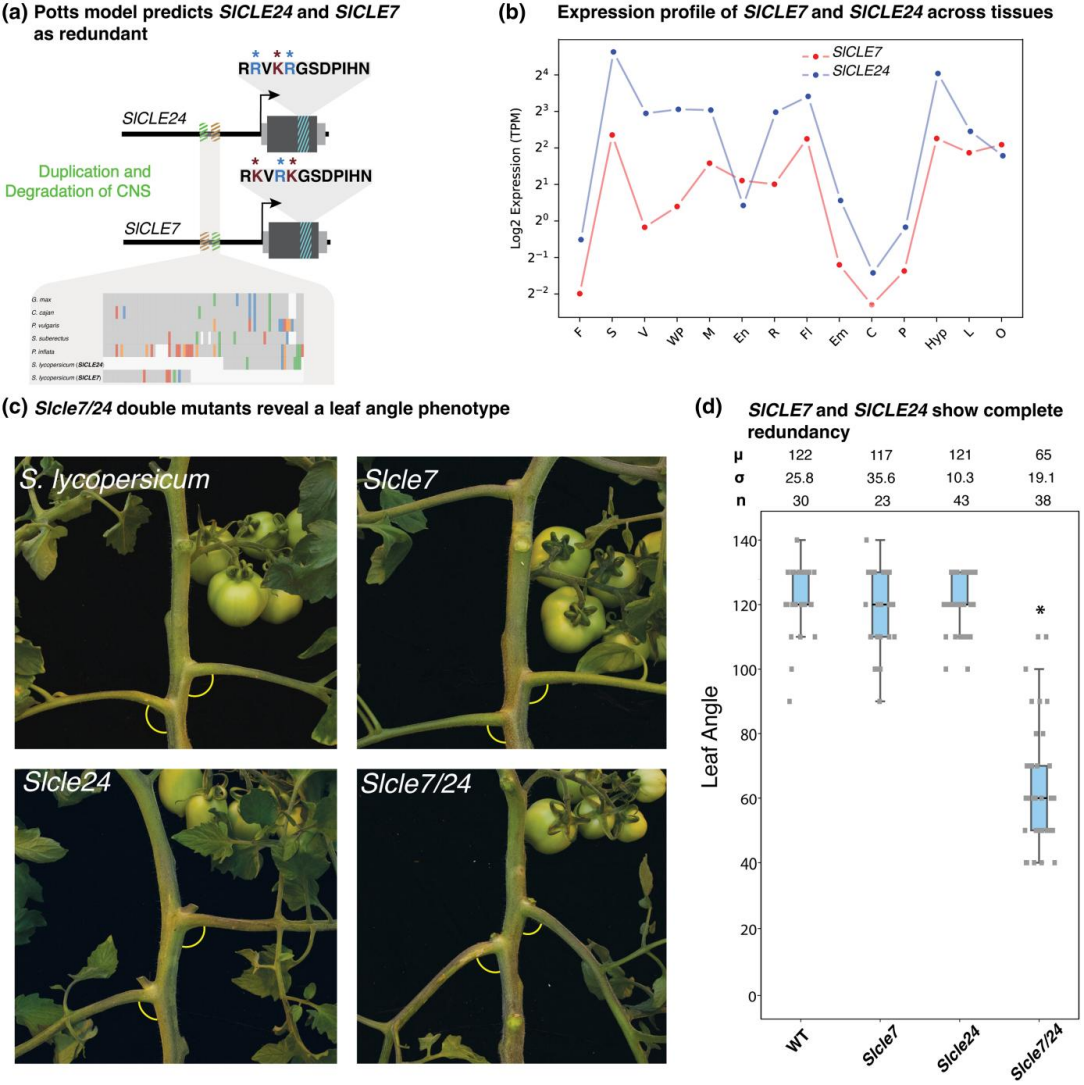
*CLE7* and *CLE24* redundantly control petiole angle in tomato. **(**a) Scheme of the variation present between *SlCLE7* and *SlCLE24*, showing sequence variation in both the CDS and the promoter region of both genes. Substitutions in the dodecapeptide with a predicted negative effect are marked in red, while positive changes are in blue. Cumulatively, the net difference between *SlCLE7* and *SlCLE24* is close to zero. The degrading promoter is represented as an alignment of a selection of species. Gray represents a match to the consensus base, while blue, red, orange, and green represent substitution to A, T, G, and C, respectively. Gaps are represented in white. *SlCLE7* and *SlCLE24* retained nonoverlapping sections of this CNS. (b) Expression (Log2 TPM) of *SlCLE7* and *SlCLE24* in different tissues (F, fruit; S, stem; V, vasculature; WP, whole plant; M, meristem; En, endosperm; R, roots; Fl, flowers; Em, embryo; C, calli; P, pollen; Hyp, hypocotyl; L, leaves; O. ovary). Red, *SlCLE7*; blue, *SlCLE24*. (c) Representative petiole angle phenotypes of WT tomato, *Slcle7* and *Slcle24* single mutants and *Slcle7 Slcle24* double mutants. (d) Quantification of petiole angle phenotype in WT tomato, *Slcle7* and *Slcle24* single mutants and *Slcle7 Slcle24* double mutants. “μ” represents the mean, “σ” refers to standard deviation, and “n” indicates the sample size used. “*” indicates a statistical difference < 0.05.

Taking into consideration both coding and promoter sequences, we can more accurately place *CLE* genes within their proper phylogenetic context, thereby enabling improved prediction and dissection of paralog relationships and divergence. Tomato *SlCLV3* and *SlCLE9* exemplify a scenario where their divergence, mediated by both amino acid substitutions and promoter degradation, has led to functional drift while preserving compensatory interactions (Kwon et al. 2022). To further explore these patterns, we focused on another pair of tomato *CLE* paralogs, *SlCLE7* and *SlCLE24*, orthologous to *AtCLE45 and AtCLE33* involved in vasculature formation (Carbonnel et al. 2023). Analysis of the *SlCLE7–SlCLE24* cluster (comprising only two members) revealed a paralog relationship mirroring that of previously dissected *SlCLV3–SlCLE9* redundancy. Despite not showing the accumulation of deleterious coding mutations between the pair, with three changes having a neutral net effect, Conservatory (Hendelman et al. 2021; Amundson et al. 2025) showed asymmetrical promoter degradation, with each paralog retaining nonoverlapping portions of a longer stretch of noncoding sequence conserved across angiosperms (Fig. 4a; see Materials and Methods). In line with this observation, *SlCLE7* and *SlCLE24* share nearly identical expression patterns across tissues, but *SlCLE24* exhibits higher expression (Fig. 4b), suggesting *SlCLE24* paralog dominance in this predicted unequal redundancy relationship (Benoit et al. 2025).

To better understand the relevance of regulatory and coding sequence conservation in the predicted redundancy relationship of *SlCLE24* and *SlCLE7*, we simultaneously mutated both genes using CRISPR/Cas9 gene editing. A screen of progeny from first-generation transgenic (T0) plants revealed a conspicuous change in leaf angle. While wild-type (WT) leaves exhibited an angle of 110°–130° relative to the main shoot, plants with null mutations in both *Slcle7* and *Slcle24* (double mutants) showed a substantially reduced leaf angle of 60°–90° (p < 0.001) (Fig. 4c and d). Notably, this reduction of leaf angle mirrors that of the previously characterized *fasciated (fas)* and *branched 2* (*fab2*) mutant (Jeong et al. 2025), defective in an enzyme involved in arabinosylation of the tomato CLV3 dodecapeptide and of other CLEs in *Arabidopsis* (Figure S8), suggesting a role for FAB2-mediated modification of *SlCLE24–SlCLE7* in this developmental syndrome (Jeong et al. 2025). This phenotype is absent in single mutants of each gene, indicating that the regulatory divergence reflected by differences in promoter sequences and gene expression is insufficient to compromise functionality; rather, the inherent strength of the peptides, as indicated by the lack of deleterious mutations, preserves their compensatory roles. In contrast to the situation observed for *SlCLV3* and *SlCLE9*, where promoter degradation significantly alters their interaction, the functional redundancy between *SlCLE7* and *SlCLE24* remains balanced, despite the observed regulatory sequence degradation and signal of paralog dominance from our sequence analysis.

### Dissection of the Complex Genetic Interactions in Two Additional CLE Clades

**Fig. 5.**
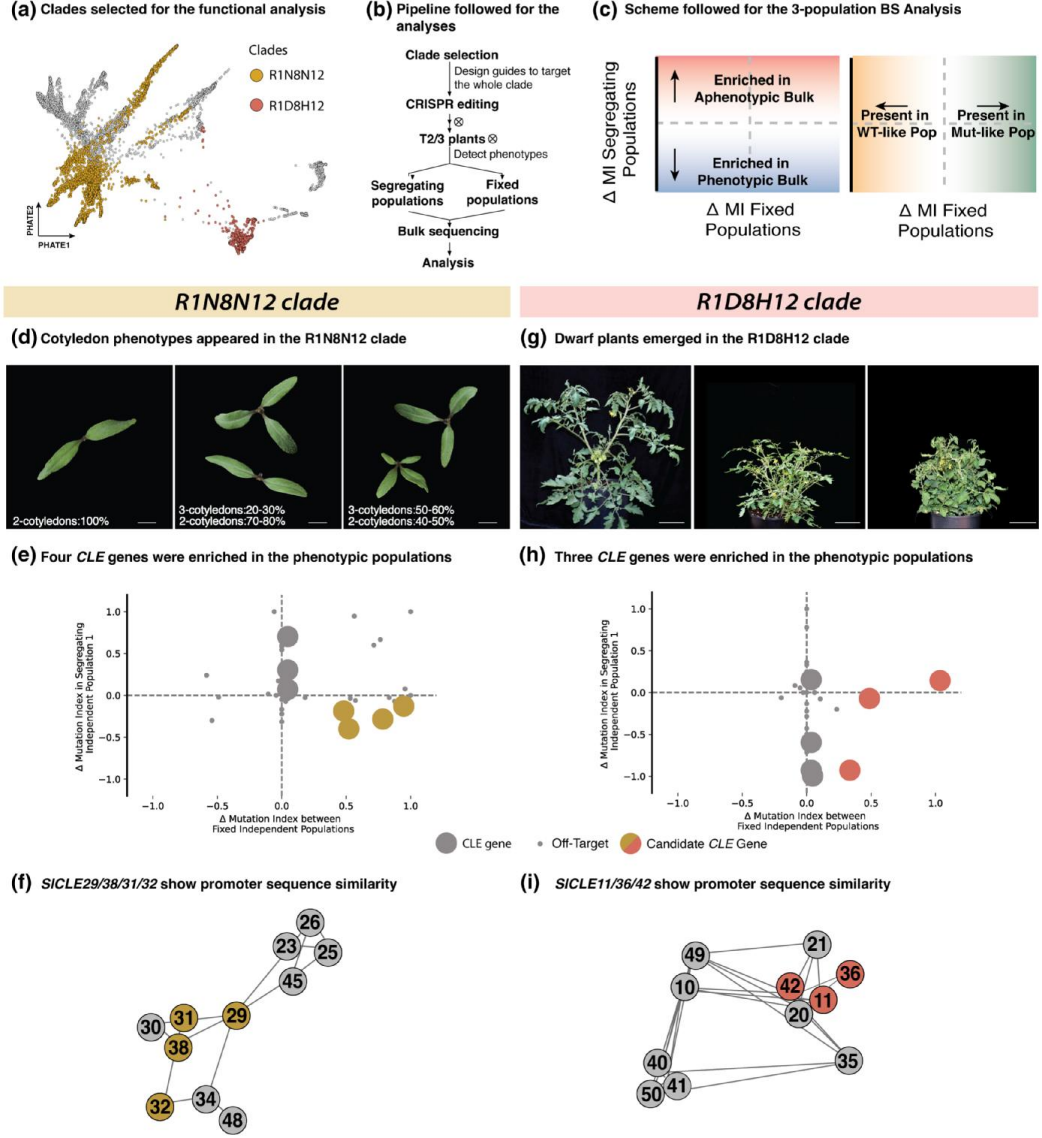
Promoter conservation correlates with *CLE* family redundancy shaping phenotypes in complex clades. (a) PHATE plot of *CLE* genes, highlighting the two clades selected for analysis, R1N8N12 in dark yellow and R1D8H12 in salmon red. (b) Pipeline to study gene relationships in complex clades, starting with CRISPR-Cas9 gene editing of the whole clade followed by phenotyping, generation of segregating and fixed populations and bulk sequencing. (c) Scheme of the output of the three-population bulk sequencing approach used to analyze candidate genes. Presence in the bottom right section indicates enrichment in the mutant phenotypic classes. MI, mutational index. (d and g) Representative phenotypes obtained by whole-clade gene editing of the two studied clades. R1N8N12 mutant plants revealed cotyledon defects with abnormal presence of extra embryonic leaves. R1D8H12 mutants revealed a more petite, compacted, and altered development compared to the aphenotypic populations. (e and h) Analysis of candidate genes from the bulk sequencing data, showing in the bottom right quarter the genes associated with the observed phenotypes. Small dots refer to off-targets, while bigger dots are *CLE* genes. (f and i) Promoter relationship network, highlighting in color the candidate genes obtained by the analysis. Each node in a promoter of a specific *CLE* gene and each edge is the similarity value between those promoters.

While *SlCLE7–SlCLE24* provides a relatively simple paralog pair to investigate how sequence divergence translates into functional drift, many other *CLE* family clades in tomato display considerably greater genetic complexity. For example, two larger *CLE* clades identified through our computational analyses, designated R1D8H12 and R1N8N12 based on amino acid composition and distribution on dendrogram (Figs 3b and 5a; Table S4), offer opportunities to dissect genetic architectures and patterns of paralog redundancy in greater depth. To account for the possibility of redundancy among multiple members of these clades, we developed a combined forward and reverse genetics approach using multiplex CRISPR/Cas9 editing that targets all members of a given clade. We generated multiple independent transgenic mutant populations and performed bulk short-read sequencing approach to genotype both segregating and fixed lines, establishing a method for associating engineered collections of mutations with emergent phenotypes (see Materials and Methods; Fig. 5b and c). This strategy addresses the limitations of simpler gene editing approaches, which often fail to resolve more complex redundancy patterns among numerous family members.

As shown in Fig. 5d, the R1N8N12 mutational pool was characterized by a multicotyledon phenotype with variable expressivity. In one population, 30% of individuals exhibited three cotyledons, whereas in another group, 50% displayed this trait, with occasional instances of four cotyledons. Notably, the *Arabidopsis* gene *AtCLE19*—a member of this superclade—has been reported to be cotyledon specific, with mutations causing cotyledon defects (Xu et al. 2015). To determine whether the tomato ortholog or other clade members contribute to this phenotype, we applied our bulk sequencing approach and identified four *CLE* genes as enriched candidates based on their combined mutational profiles (*SlCLE29*, *SlCLE31*, *SlCLE32*, and *SlCLE38*) (Fig. 5e; Tables S6, S8, and S9 for genotyping information). Importantly, analysis of our integrated datasets, encompassing mutation distances and noncoding sequence information, revealed that promoter similarity predominantly explains the high-order mutational patterns and associated phenotypes observed in the CRISPR lines. Indeed, the top candidate genes clustered tightly within the promoter similarity network, showing a statistically significant pattern that would not occur by chance (Fig. 5f).

A similar pattern was observed for the R1D8H12 clade, where plants from different populations exhibited vegetative defects characterized by thinner stems and less complex plant architecture with variable severity (Fig. 5g). Bulk whole-genome Illumina sequencing of these populations revealed enrichment of *SlCLE11*, *SlCLE36*, and *SlCLE42* (Fig. 5h; Tables S7, S8, and S10 for genotyping information). Intriguingly, like the putative causal mutations from the R1N8N12 clade, these three genes clustered closely in the promoter similarity network rather than by ME variation and expression that on the other hand show contradicting patterns, including for *SlCLE11* that shows the strongest expression profile and the peptide with the highest amount of deleterious mutations (Fig. 5i; Figure S9a and b).

### Summary

In this study, we employed an interdisciplinary approach, from custom de novo gene-annotation pipeline to CRISPR mutagenesis, to decode the diversification of CLE signaling peptides across flowering plants. This strategy not only yielded broad insights into *CLE* family evolution but also established a framework for studying other rapidly evolving gene families and for dissecting the coevolutionary dynamics including between their interacting partners, for example, the reciprocal changes between CLE peptides and their LRR receptors. By bridging the gap between model and nonmodel species our deep pan-genomic sampling, empowered by over 2,000 angiosperm genomes, allowed us to disentangle the relative contributions of changes in coding versus cis-regulatory sequences in the diversification of paralogs and their redundancies. By juxtaposing *SlCLE7/24*, whose nearly invariant coding sequences underpin a classical redundancy, with the R1N8N12 and R1D8H12 clades, in which highly conserved promoters rather than protein identity sustain overlapping functions, we expose two complementary routes by which paralogs buffer development.

Our study demonstrates that a large number of genomes spanning a broad evolutionary space can improve predictions on gene divergence happening over short timescales. Indeed, through comparative functional analyses of the *CLV3* clade in Solanaceae (tomato, forest nightshade, and ground-cherry), representing less than 50 million years of evolution (J. He et al. 2023), we found that phenotypes derived from CRISPR-mediated base-editing of the dodecapeptide aligned with predicted ME modeled based on the full sequence diversity of this gene family. This demonstrated that global modeling of gene family sequence diversity can predict local patterns of its genotype–phenotype landscape among cohorts of paralogs within those gene families. The phenotypic effect of mutations is the mechanism by which gene families expand and fine-tune their functional repertoire while preserving essential roles. By integrating deep comparative genomics with predictive mutational modeling and targeted editing, we provide a roadmap for forecasting when redundancy will fail, how compensation evolves, and how hidden paralog variation can be leveraged to reshape plant form and function.

## Materials and Methods

### CLE and LRR Discovery and de Novo Annotation

All previously annotated CLE genes were searched in available resources of the Conservatory project (Hendelman et al. 2021; Amundson et al. 2025), which collected the genomes of more than 300 species, assessed to have a complete annotation. This dataset was then used as a query to search using Diamond (Buchfink, Xie, and Huson 2015) in all the available plant genomes on NCBI with an assembly status of Scaffold or Chromosome [Diamond parameters: –ultra-sensitive –masking 0 –iterate]. The identified hits were isolated, including 1,000 bp upstream and downstream (customized Julia script). These sequences were subsequently analyzed by MAKER2 (Holt and Yandell 2011) with dynamic parametrization based on the genome of origin. The predicted proteins were then assessed for being true CLE genes by checking for the presence of a signaling peptide and the CLE motif using InterProScan and HMMER [hmmsearch with parameters: hmmsearch –max -T 0] (Jones et al. 2014; Finn, Clements, and Eddy 2011). The newly discovered CLEs were then compared with BEDTools (Quinlan and Hall 2010) to the original source of the well-curated annotation files screened by the Conservatory project to evaluate whether the developed pipeline captured previously unknown genes.

For LRR genes, we run HHMER [hmmsearch with parameters: hmmsearch –max -T 0] for all the proteins from the Conservatory genomes. We then filtered for those proteins with >10 LRRs and a kinase domain.

All scripts mentioned above are available on https://github.com/LippmanLab/Pan_angiosperm_CLE_annotation.

### Sequence Similarity Hierarchical Analysis and Conservation Analysis

We generated an all-by-all reciprocal BLASTp comparison using CLANS 2.0 (Frickey and Lupas 2004, available on CLANS2.0 https://github.com/inbalpaz/CLANS). We constructed an undirected network using − log10 (BLASTp E-value) as edge weight. We then pruned the network so that each node maintained the top 500 connections (customized Python script). Node2Vec (Grover and Leskovec 2016), implemented in SNAP (Leskovec and Sosic 2016), was then used to vectorize the outputted graph [-l:200 -r:600 -k:100 -e:1 -w]. The vectorized network in 128 dimensions was then projected into a lower-dimensional space using PHATE (Moon et al. 2019). To detect paralogy and homology, we implemented the Leiden algorithm (customized Python script). We adopted multiple increasing resolution parameters and reconciled all the outputs into a hierarchical structure using the Multi-resolution Reconciled Tree in R (Peng et al. 2021). To assess and resolve the overclustering at the leaf level in the generated tree, we implemented a RandomForest-based permutation test (similar to the system used in CHOIR (Petersen et al. 2025)) using the dodecapeptide as a 12-dimensional label space. Sister leaves that failed the permutation test were pruned. To resolve the polytomy at higher nodes, we adopted a parsimonious entropy minimization test based on the dodecapeptide amino acid composition (customized Python script). To integrate promoter information into the structure to better delineate paralogy and homology, we first constructed a kNN graph based on the vectorized BLASTp network. Each node was unlabeled except for those CLE genes that overlapped with the Conservatory project. We extracted from the Conservatory project the promoter conservation levels with other CLE genes within the same genome or between species. CLE genes with shared promoters were labeled as a joint or the group and labeled accordingly. To sort the remaining CLE genes that did not have promoter information available, we applied a label propagation algorithm to the partially annotated kNN graph (customized Python script).

For LRR gene classification, we took previously published and analyzed sequences (Man et al. 2023) and classified them in their specific phylogenetic classes and built a random-forest classifier for each phylogenetic clade based on pairwise comparison. We then applied this classification model to the discovered sequences in this study and overlapped the distributions of these predicted labels to the outputs of Leiden clustering, observing clear overlaps.

For the conservation analysis via EMS2, we adopted the method in Yeung et al. (2023) (model esm2_t33_650M_UR50D). The conservation score was calculated as the proportion of the positions with a site-level value above 0.6.

For MMseq2, we used default options and converted the output to an edge list using a customized script.

All above scrips are available at https://github.com/LippmanLab/Pan_angiosperm_CLE_annotation.

### ME Estimation and Validation

To assess the mutational burden between paralogs or homologs, we extracted all the CLE motifs from our dataset based on the match derived from hmmsearch. The subsequent list of sequences was treated as a gapless multiple sequence alignment and inputted in EVmutation (Hopf et al. 2017) following the author's guidelines and implemented in pipeline available on GitHub. The derived results were validated computationally by focusing on SlCLV3. The three-dimensional structure of SpriCLV3 interacting with its LRR receptor SpriCLV1 was obtained using AlphaFold-Multimer (default parameter—relaxed) (AlphaFold-Multimer). The generated structure was cross-validated with known information about the physical interaction between CLE peptides and LRR receptors (Morita et al. 2016). To validate AlphaFold-Multimer, AlphaFold3 was used using default options on the AlphaFold3 server (Abramson et al. 2024). For both models, the extracellular domain of SpriCLV1 was used (sequence details in the json file generator code). Once the structure was validated, confirming the power of AlphaFold-Multimer to generate outputs resembling the expected interactions, we performed an in silico saturation mutagenesis using SSIPe (Huang et al. 2020) to evaluate binding affinity and AlphaFold-Multimer to assess docking, following the method described in Yang et al. (2022). In addition to these measurements, ME values were also compared to the biochemical properties of the substitutions (Sneath index). The resulting quantifications were used in a regression analysis to evaluate our predicted MEs. For the molecular dynamics emulations, we followed the method developed in Lewis et al. (2025) using SpriCLV3 as tested peptides. The mentioned scripts are available at https://github.com/LippmanLab/Pan_angiosperm_CLE_annotation.

### Plant Materials, Growth Conditions, and Phenotyping

As previously described in Ciren et al. (2024), seeds of WT *S. lycopersicum* (cultivar M82, LA3475), *Solanum prinophyllom*, and *Physalis grisea* (ZL05) were used. Seeds were directly sown in soil in 96-cell plastic flats and grown to 4-week-old seedlings in the greenhouse. Seedlings were then transplanted to 4 L pots in the greenhouse for crossing and bulking purposes or directly to the fields at Cold Spring Harbor Laboratory, New York. Greenhouse conditions are long-day (16 h light, 26-28 °C/8 h dark, 18-20 °C; 40%-60% relative humidity) with natural light supplemented with artificial light from high pressure sodium bulbs (250 umol m−2·s−1). Plants in the fields were grown under drip irrigation and standard fertilizer regimes and were used for quantifications of inflorescence branching, sepal length, and fruit shape. Quantitative phenotypic data were collected manually in fields and greenhouses. Raw leaf angle data are in Table S12.

### Expression Analysis

Publically available RNA-seq datasets for tomato and other species were used (Table S11). For tomato, a similar approach used in Benoit et al. (2025) was implemented: Raw reads were realigned to the reference genome and transcripts per million (TPM) quantified. Only samples with more than 50% uniquely mapped reads were retained for subsequent analysis. Further filtering was applied based on the Spearman correlation between tissue replicates and removed samples with low correlation (0.75 or below).

### Genome Editing

As previously described in Ciren et al. (2024), CRISPR/Cas9 mutagenesis and generation of transgenic tomato plants were performed following our standard protocol. Briefly, guide RNAs (gRNAs) (listed in Table S9) were designed using the Geneious Prime software. For Cas9 multiplex editing, the Golden Gate cloning system was used to assemble the binary vector containing the Cas9 and the specific gRNAs. For base-editing, vectors were constructed through a modular GatewayTM assembly, as described previously (Invitrogen). Final binary vectors were then transformed into the tomato cultivar M82 by *Agrobacterium tumefaciens*-mediated transformation through tissue culture. Regenerated plants and first-generation plants were genotyped in the target regions through primers designed in the Geneious Prime software (listed in Table S9).

### Three-Population Analysis

As described, second- and third-generation transgenic plants (T2/3) were genotyped via Illumina-based WGS on NextSeq 2000 P3 sequencing platform (Illumina). Plants showing a phenotype were bulked together and sequenced. Reads were aligned to the M82 Genome (Alonge et al. 2022) via BWA (Li and Durbin 2010). Polymorphisms were called with SnpSift (Cingolani et al. 2012) (Table S7 for *CLE* gene-specific alleles and Table S8 for the entire SnpSift output) and analyzed in a customized Python script based on comparison of a segregating population against two fixed populations, wt-like and mutant-like.

## Supplementary Material

msaf294_Supplementary_Data

## Data Availability

All alignments, trees, rate test results, selection test results, and phenotyping data are available on the Lippman lab's GitHub (https://github.com/LippmanLab/Pan_angiosperm_CLE_annotation).
