# Supplementary material for: Pan-Angiosperm Analysis of the CLE Signaling Peptide Gene Family Unveils Paths, Patterns, and Predictions of Paralog Diversification
Source: Mol Biol Evol. 2025 Nov 13;42(11):msaf294. doi: 10.1093/molbev/msaf294 (PMC12661658; doi:10.1093/molbev/msaf294)
Supplement: msaf294_Supplementary_Data [file msaf294_supplementary_data.zip › Sup_figures.pdf]

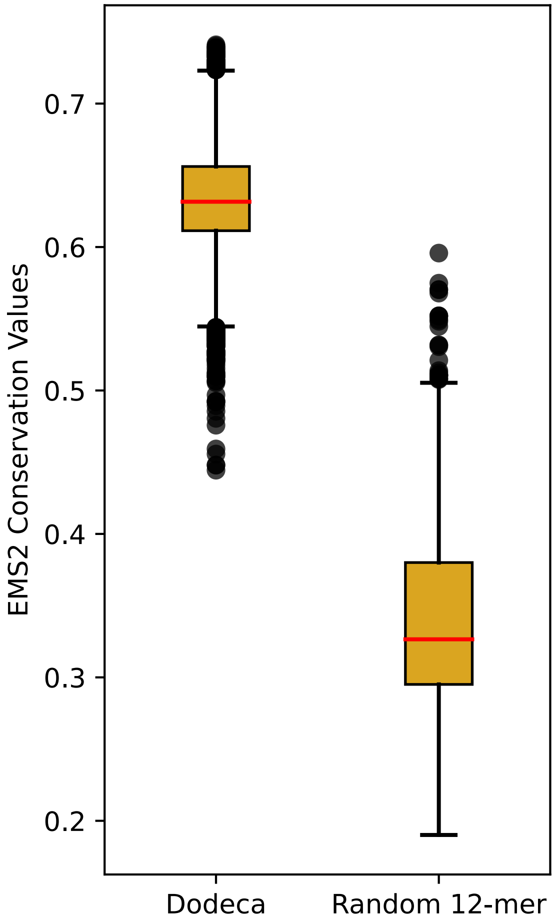

Supplementary Figure 1. EMS2 Model recognizes CLE Dodecapeptides. The outputted Conservation Value from EMS2 is higher for the section of the CLE protein encoding for the functional 12-mer motif.

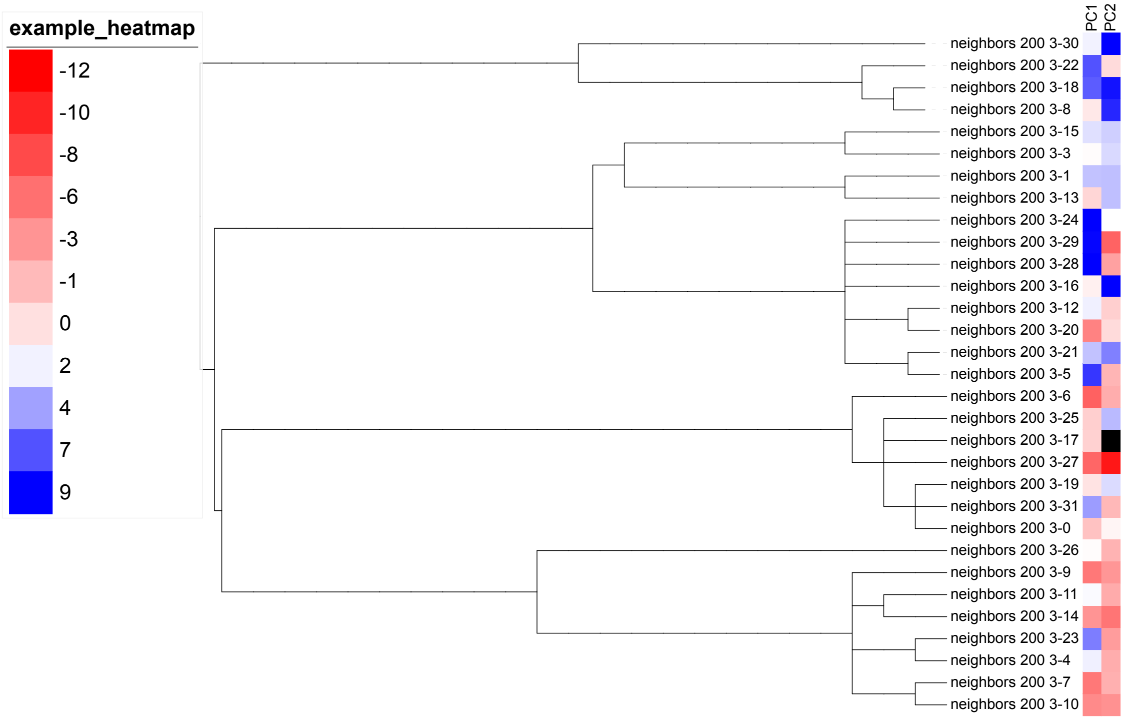

Supplementary Figure 2. Golgi Signalling peptide sequence Composition in CLE genes. PC1 and PC2 values for Golgi Signalling peptide sequence composition reveals patterns aligning with the structure of the inferred hierarchical sequence relationship.

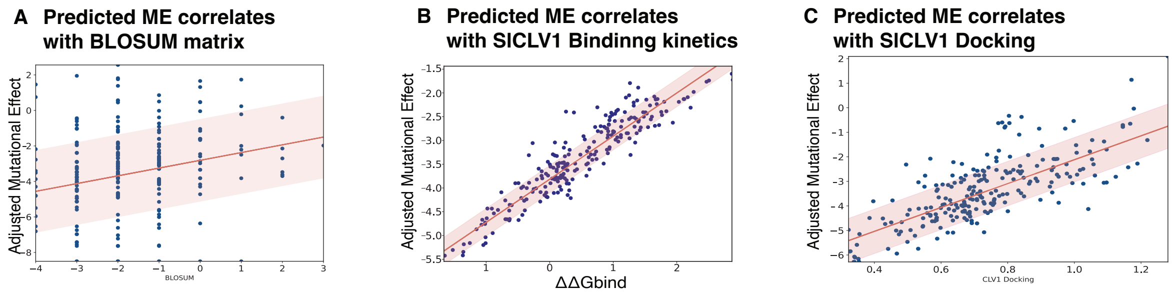

Supplementary Figure 3. Mutational Effects correlate with relevant biological aspects of CLE biology. A) Mutational Effect prediction correlates with BLOSUM substitution matrix. B) Mutational Effect prediction correlates with Kinetics values derived from SSIPe. C) Mutational Effect prediction correlates with Alphafold Docking scores.

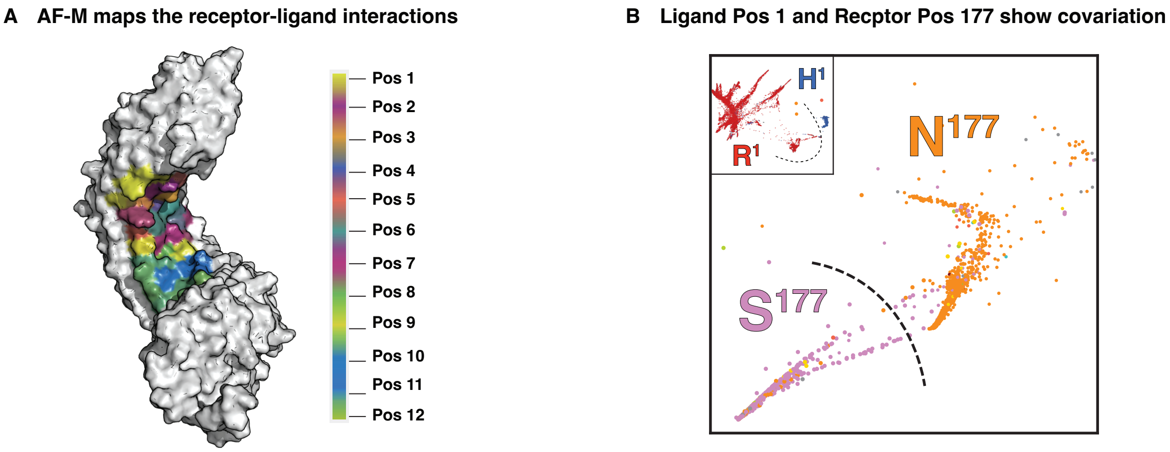

Supplementary Figure 4. Our pipeline enables new analysis of co-evolutionary dynamics between CLE genes and LRR receptors. A) Alpha-Fold Multimer structure of CLE receptors, highlighting the consensus CLE binding surface per residue derived from aggregated simulations with all the possible CLE dodecapeptides. B) PHATE plot of the CLV1-BAM-PXY receptor genes and CLE genes showing a similar covariation pattern between ligand position 1 (H/R) and receptor position 177 (N/S).

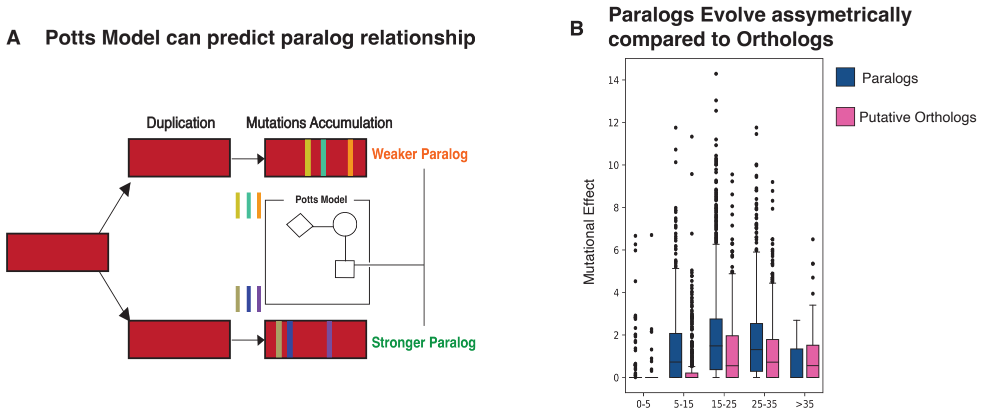

Supplementary Figure 5. Paralogs have a higher Mutational Burden than Singletons. A) By treating paralogs as the same gene with differentiating mutations, the trained Potts model can interpret these amino acid changes and determine which paralog accumulated the most deleterious amino acid changes. B) Overall, within-species paralog comparisons have a higher mutational burden than between-species singletons.

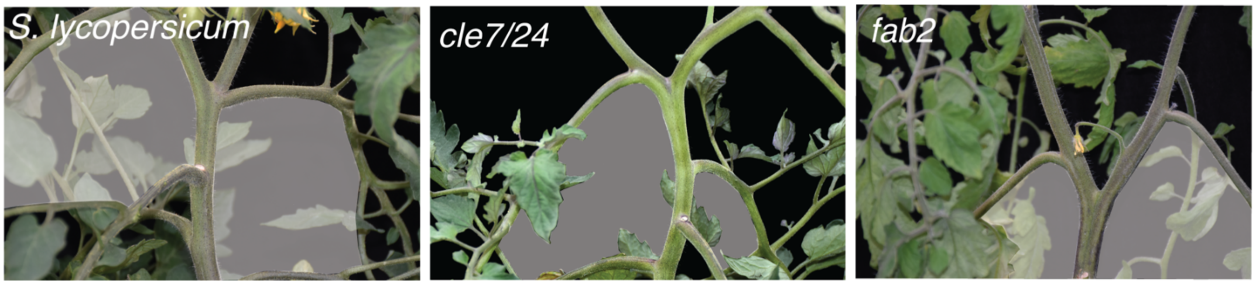

Supplementary Figure 6. *cle7/24* mutant phenocopies *fab2* mutant, an enzyme involved in the post-translational modification of these peptides.

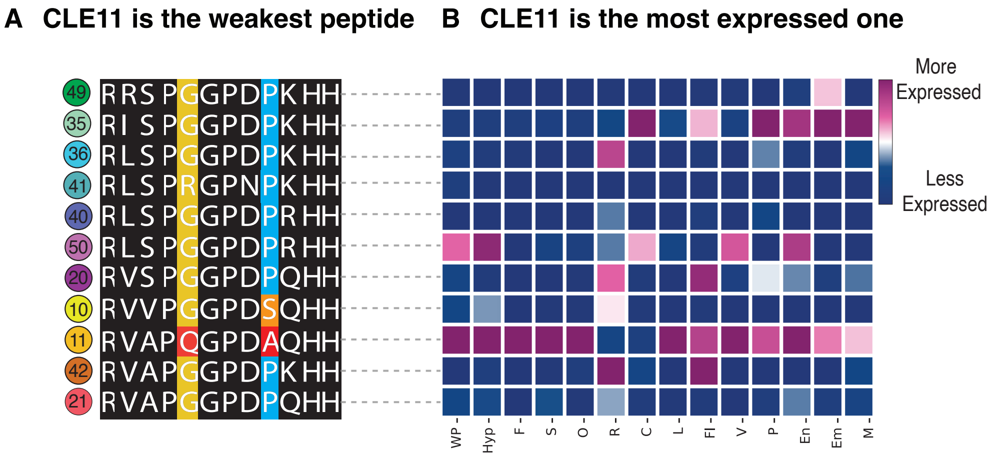

Supplementary Figure 7. Protein and Expression Analysis of the R1D8H12 Clade. A) Their dodecapeptide composition does not show major variability, except for CLE11 having key amino acid substitutions occurring in two relevant positions. B) Despite being the weakest peptide, CLE11 is the member of this clade showing the highest level of expression, except in roots where CLE42 is the highest expressed gene.
