# Supplementary material for: Pan-Angiosperm Analysis of the CLE Signaling Peptide Gene Family Unveils Paths, Patterns, and Predictions of Paralog Diversification
Source: Mol Biol Evol. 2025 Nov 13;42(11):msaf294. doi: 10.1093/molbev/msaf294 (PMC12661658; doi:10.1093/molbev/msaf294)
Supplement: msaf294_Supplementary_Data [file msaf294_supplementary_data.zip › Sup_figures_R_round2.docx]

**Supplementary Figure 1. Technical assessments of methods used.** A**)** EMS2 Model recognizes CLE dodecapeptides. The outputted Conservation. The outputted Conservation Value from EMS2 is higher for the section of the CLE protein encoding for the functional 12-mer motif. B) Distance analysis among points in the embedding space generated by Node2Vec and the cartesian space generated by CLANS (FR) given an increasing number of neighbors. CLANS shows a strong distortion with closely related proteins being compressed in a defined space with abrupt transition among clusters.

**Supplementary Figure 2. Golgi Signalling peptide sequence Composition in CLE genes.** PC1 and PC2 values for Golgi Signalling peptide sequence composition reveals patterns aligning with the structure of the inferred hierarchical sequence relationship.

**Supplementary Figure 3. MMseq2 based similarity network represent a parsimonious distribution of aminoacid changes, in line with BLASTp.** PHATE Map of the MMseq2 pairwise comparison network of *CLE* genes showing the different aminoacid changes for each position

**Supplementary Figure 4. Our pipeline enables new analysis of co-evolutionary dynamics between CLE genes and LRR receptors.** A) Alpha-Fold Multimer structure of CLE receptors, highlighting the consensus CLE binding surface per residue derived from aggregated simulations with all the possible CLE dodecapeptides. B) PHATE plot of the CLV1-BAM-PXY receptor genes and CLE genes showing a similar covariation pattern between ligand position 1 (H/R) and receptor position 177 (N/S).

**Supplementary Figure 5. Potts Model aminoacidic co-evolutionary map correlates with physical interaction.** A) Contact map derived from Potts model representing the level of co-variation. B) Frequency of physical interaction between positions in molecular dynamics emulations.C) Co-variation patterns correlate with physical interactions

**Supplementary Figure 6. Mutational Effects correlate with relevant biological aspects of CLE biology.** A) Mutational Effect prediction correlates with BLOSUM substitution matrix. B) Mutational Effect prediction correlates with Kinetics values derived from SSIPe. C) Mutational Effect prediction correlates with Alphafold Docking scores. D) Correlation between AFM and AF3 scores

**Supplementary Figure 7. Paralogs have a higher Mutational Burden than Singletons.** A) By treating paralogs as the same gene with differentiating mutations, the trained Potts model can interpret these amino acid changes and determine which paralog accumulated the most deleterious amino acid changes. B) Overall, within-species paralog comparisons have a higher mutational burden than between-species singletons.


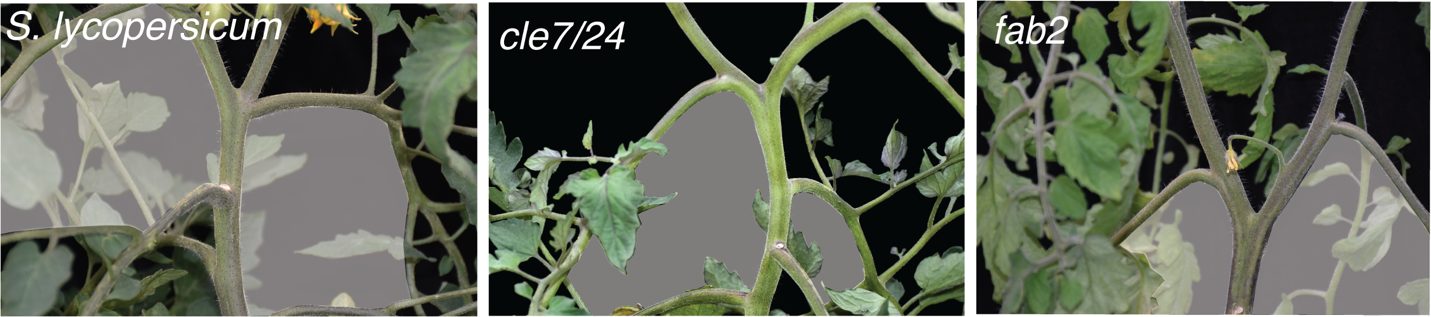


**Supplementary Figure 8. cle7/24 mutant phenocopies fab2 mutant, an enzyme involved in the post-translational modification of these peptides.** WT tomato (S. lycopersicum) do not show any defects in leaf angle, while cle7/24 and fab2 show wilting effect.

**Supplementary Figure 9. Protein and Expression Analysis of the R1D8H12 Clade.** A) Their dodecapeptide composition does not show major variability, except for CLE11 having key amino acid substitutions occurring in two relevant positions. B) Despite being the weakest peptide, CLE11 is the member of this clade showing the highest level of expression, except in roots where CLE42 is the highest expressed gene.
